# The Roles of Carotenoid Consumption and Bioavailability in Cardiovascular Health

**DOI:** 10.3390/antiox10121978

**Published:** 2021-12-11

**Authors:** Yuanhang Yao, Hongyi Manfred Goh, Jung Eun Kim

**Affiliations:** Department of Food Science and Technology, National University of Singapore, Singapore 117543, Singapore; yuanhang@u.nus.edu (Y.Y.); manfred_goh@u.nus.edu (H.M.G.)

**Keywords:** carotenoids, cardiovascular disease, oxidative stress, inflammation, vascular health, bioavailability

## Abstract

Carotenoids are natural pigments generally with a polyene chain consisting of 9–11 double bonds. In recent years, there has been increasing research interest in carotenoids because of their protective roles in cardiovascular diseases (CVDs). While the consumption of carotenoids may have a beneficial effect on CVDs, the literature shows inconsistencies between carotenoid consumption and reductions in the risk of CVDs. Therefore, this review aims to provide a summary of the association between dietary carotenoid intake and the risk of CVDs from published epidemiological studies. Meanwhile, to further elucidate the roles of carotenoid intake in CVD protection, this review outlines the evidence reporting the effects of carotenoids on cardiovascular health from randomized controlled trials by assessing classical CVD risk factors, oxidative stress, inflammatory markers and vascular health-related parameters, respectively. Given the considerable discrepancies among the published results, this review underlines the importance of bioavailability and summarizes the current dietary strategies for improving the bioavailability of carotenoids. In conclusion, this review supports the protective roles of carotenoids against CVDs, possibly by attenuating oxidative stress and mitigating inflammatory response. In addition, this review suggests that the bioavailability of carotenoids should be considered when evaluating the roles of carotenoids in CVD protection.

## 1. Introduction

Cardiovascular diseases (CVDs) are the leading cause of death globally, accounting for approximately 32% of all deaths [1]. The World Economic Forum and Harvard School of Public Health [2] estimate that the total economic cost of CVDs, arising from both direct healthcare cost and the loss of productivity, could increase from $863 billion in 2010 to $1044 billion in 2030. Given the high mortality rate and alarming economic cost, understanding the risk factors for CVDs is of paramount importance.

CVDs refer to a myriad of disorders involving the heart and blood vessels. This includes coronary heart disease (CHD), cerebrovascular disease, peripheral vascular disease, rheumatic heart disease, congenital heart disease, deep vein thrombosis and pulmonary embolism [1]. While the deposition of lipids on the endothelium of the blood vessels of the brain and heart is the most common cause of CVDs, recent research has suggested that oxidative stress and inflammation are key contributors to the pathogenesis of CVDs [3]. For instance, excessive production of reactive oxygen species (ROS) is known to induce cellular abnormality within the vasculature that may eventually contribute to cardiac dysfunction [4]. In particular, the oxidation of low-density lipoprotein (LDL) by ROS in the vessels plays a key role in the development of atherosclerosis [5]. Inflammatory processes are also known to be involved in the development of atherosclerosis [6].

According to the 2013 American College of Cardiology and the American Heart Association guidelines [7], lifestyle modification is key in CVD prevention. Among these lifestyle modification guidelines, the adoption of a healthy diet is one of the most important strategies. In particular, a diet that emphasizes the consumption of fruits and vegetables is promoted for its beneficial effects on CVD risk reduction [8] and this may be attributed to carotenoids, one of the nutrients found in fruits and vegetables.

Carotenoids are some of the most abundant natural pigments, with more than 700 different compounds having been identified so far [9]. However, only about 50 compounds are found in the diet that can be absorbed and utilized by humans [10]. Carotenoids are divided into two classes: xanthophylls (containing oxygen) and carotenes (pure hydrocarbons that do not contain oxygen). Xanthophylls include lutein, zeaxanthin and β-cryptoxanthin, while carotenes include β-carotene, lycopene and α-carotene. These compounds are responsible for the red, orange and yellow colors found in plants and some animals [11]. Characterized by their extended π-electron conjugated system comprising isoprene units [9], carotenoids are able to exhibit antioxidant properties by directly quenching singlet oxygen or by reacting with free radicals [12]. The antioxidant activity of carotenoids is regarded to be a viable property for the prevention and melioration of CVDs [13].Beyond its antioxidant properties, carotenoids may interact with cellular signalling pathways, suppressing oxidative stress and inflammatory processes [9].

While carotenoids may have beneficial effects on CVDs, the association between higher carotenoid intake and the lower risk of CVDs is still controversial due to conflicting findings [14,15,16,17]. However, an inverse relationship between plasma carotenoid concentrations and the risk of CVDs was validated by several epidemiological studies [18,19,20,21]. As such, bioavailability may act as a confounding factor and is of importance when evaluating the roles of carotenoids in CVD protection. This review aims to provide a summary of the published results from epidemiological and interventional studies regarding the potential roles of carotenoids in CVD protection. In addition, the dietary factors affecting the bioavailability of carotenoids will be also discussed.

## 2. Observational Studies on Carotenoids and CVDs

Although many epidemiological studies have investigated the association between carotenoid consumption and the risk for various CVDs, the results of these studies have been inconsistent. Table 1 below summarizes the results of observational studies that have investigated the relationship between the intake of individual and total carotenoids and the risk of CVDs.

### 2.1. Lutein, Zeaxanthin and β-Cryptoxanthin

In the Alpha-Tocopherol, Beta-Carotene Cancer Prevention study, Hirvonen et al. [14] concluded that there was a significant inverse association between lutein plus zeaxanthin intake and the risk of subarachnoid hemorrhage (risk ratio (RR): 0.47, 95% confidence interval (CI): 0.24–0.93) in a multivariate analysis adjusted for age, supplementation group, blood pressure and other risk factors when comparing the highest and the lowest quartile intake. However, the association was no longer significant after the model was simultaneously adjusted for other dietary antioxidants. In addition, no significant association between lutein plus zeaxanthin intake and the risk of other forms of stroke (cerebral infarction and intracerebral hemorrhage) was noted. 

However, from the Nurses’ Health study, Osganian et al. [15] reported that there was no association between the highest quintile of lutein plus zeaxanthin intake and coronary artery disease (CAD) risk (RR: 0.90, 95% CI: 0.72–1.12) as compared to the lowest quintile in a multivariate analysis adjusted for confounding factors such as age, smoking and other CVD risk factors. Similarly, Tavani et al. [22] reported a non-significant inverse association in the odds of acute myocardial infarction (AMI) with lutein plus zeaxanthin intake when comparing the highest and lowest quartile intakes (Odds Ratio (OR): 0.71, 95% CI: 0.50–1.01) in an unconditional multiple logistic regression model adjusted for age, sex, study, education and other risk factors.

In addition to lutein and zeaxanthin, β-cryptoxanthin is another common type of xanthophyll that is rich in the diet. In the Milan non-fatal AMI case–control study, Tavani et al. [22] observed a significant inverse association in the odds of AMI with increasing intake of β-cryptoxanthin when comparing the highest and lowest quartile intakes (OR: 0.64, 95% CI: 0.46–0.88) in an unconditional multiple logistic regression model including age, sex, study, education and other CVD risk factors.

### 2.2. α-Carotene

Osganian et al. [15] conducted a prospective cohort study to investigate the relationship between the dietary intake of various carotenoids and the risk of CAD in 73,286 female nurses in the Nurses’ Health Study from year 1984 to 1996. In the 12 years of follow-up, the authors identified 998 incidences of CAD. A significant inverse association between the highest quintile α-carotene intake (referenced to the lowest quintile intake) and the risk of CAD was observed in a multivariate analysis adjusted for confounding factors, including age, smoking and other CAD risk factors (RR: 0.80, 95% CI: 0.55–0.99). Similarly, in the Milan non-fatal AMI case–control study comprising 760 patients with non-fatal AMI and 682 controls, Tavani et al. [22] reported a significant inverse association in the odds of AMI with increasing intake of α-carotene when comparing the highest and lowest quartile intakes in an unconditional multiple logistic regression model, including terms for age, sex, study, education and various AMI risk factors. 

### 2.3. β-Carotene

The associations between the dietary intake of β-carotene and CVDs have been conflicting. In the prospective cohort study with 6.1 years of follow-up involving 26,593 Finnish male smokers, Hirvonen et al. [14] reported a significant inverse association between β-carotene intake and the risk of cerebral infarction (RR of highest quartile compared to the lowest quartile: 0.74, 95% CI: 0.60–0.91), even after simultaneous modeling for other antioxidants (RR: 0.77, 95% CI: 0.61–0.99). Similarly, in the Northern Italy AMI female study involving 433 cases of females with non-fatal AMI and 869 controls, Tavani et al. [23] highlighted a significant inverse association between β-carotene intake and the odds of AMI when comparing the highest quintile of β-carotene intake to the lowest (OR: 0.5, 95% CI: 0.3–0.8) in an unconditional multiple logistic regression adjusted for various confounding variables. In the Rotterdam study, Klipstein-Grobusch et al. [24] conducted a prospective cohort study involving 4,802 elderly Rotterdam residents aged ≥55 followed-up for 4 years. The authors reported that β-carotene intake was significantly and inversely associated with the risk of MI when comparing the highest tertile of β-carotene intake to the lowest in a multivariate logistics regression model adjusted for age, sex, body mass index, cigarette pack years, income, education, alcohol consumption, energy-adjusted intake of vitamins C and E and other antioxidant vitamin supplementation (RR: 0.55, 95% CI: 0.34–0.83). This was corroborated by Osganian et al. [15], who reported a significant inverse association between the highest quintile of β-carotene intake and the risk of CAD.

However, in the Chicago Western Electric Study, a prospective cohort study involving 1843 middle-aged men, Daviglus et al. [25] reported no association between the dietary intake of β-carotene (comparing the highest and the lowest quartile) and the stroke incidence (RR: 0.84, 95% CI: 0.57–1.24) when adjusted for CVD risk factors. A similar trend was observed for stroke mortality as well. McQuillan et al. [26] published the findings in year 1997 from a cross-sectional study involving 558 middle-aged males and 553 middle-aged females from the Perth Carotid Ultrasound Disease Assessment Study cohort, investigating the relationship between dietary β-carotene intake and the common carotid artery intima–media thickness (IMT) and the presence of focal plaque. No significant association between β-carotene intake and carotid artery IMT or the presence of focal carotid artery plaque was observed either with males or females in a multivariate analysis adjusted for age and conventional risk factors. In the Milan non-fatal AMI case–control study, Tavani et al. [22] also reported no association between the odds of AMI and the β-carotene intake when comparing the highest and lowest quartile intakes of β-carotene in an unconditional multiple logistic regression model including various confounding variables (OR: 0.71, 95% CI: 0.50–1.01). In year 1995, Pandey et al. [27] published their findings in a prospective study with 24 years of follow-up involving 1,556 middle-aged males from the Western Electric Company Study. The authors reported that an increase of 3 mg β-carotene intake was not significantly associated with a decrease in coronary disease mortality (RR: 0.79, 95% CI: 0.60–1.04).

**Table 1:** Association between the dietary intake of carotenoids and the risk of CVDs from observational studies.

Carotenoids	Study Design	Subjects	Outcome	Main Results	Reference
Lutein, zeaxanthin and β-cryptoxanthin	Prospective cohort study	73,286 female nurses aged 30–55	CAD incidences	No significant association was found between the highest quintile of lutein plus zeaxanthin intake and the CAD risk.	[15]
	Prospective cohort study	26,593 Finnish male smokers aged 50–69	Stroke incidences	An inverse association was found between lutein plus zeaxanthin intake and the risk of subarachnoid hemorrhage. No significant association was found between lutein plus zeaxanthin intake and the risk of all forms of stroke.	[14]
	Case–control study	Cases: 760 patients with non-fatal AMI; Control: 682 patients	AMI	No association was found in the adjusted odds of AMI with increasing lutein plus zeaxanthin intake when comparing the highest and the lowest quartile intakes.	[22]
	Case–control study	Cases: 760 patients with non-fatal AMI; Control: 682 patients	AMI	An inverse association was found in the adjusted odds of AMI with increasing intake of β-cryptoxanthin when comparing the highest and the lowest quartile intakes.	[22]
α-carotene	Prospective cohort study	73,286 female nurses aged 30–55	CAD incidences	An inverse association was found between the highest quintile of α-carotene intake and the CAD risk.	[15]
	Case–control study	Cases: 760 patients with non-fatal AMI; Control: 682 patients	AMI	An inverse association was found in the adjusted odds of AMI with increasing intake of α-carotene when comparing the highest and the lowest quartile intakes.	[22]
β-carotene	Prospective cohort study	73,286 female nurses aged 30–55	CAD incidences	An inverse association was found between the highest quintile of β-carotene intake and the CAD risk.	[15]
	Prospective cohort study	26,593 Finnish male smokers aged 50–69	Stroke incidences	An inverse association was found between β-carotene intake and the risk of cerebral infarction.	[14]
	Prospective cohort study	1,843 middle-aged males	Stroke incidences and mortality	No association was found between β-carotene intake and the risk of stroke when comparing the highest and the lowest quartile intakes.	[25]
	Case–control study	Cases: 433 females with nonfatal AMI; Control: 869 females in hospital	Non-fatal AMI	An inverse association was found between β-carotene intake and the odds of AMI when comparing the extreme quintiles of intake.	[23]
	Prospective cohort study	4,802 Rotterdam residents aged ≥55	MI	An inverse association was found between β-carotene intake and the risk of MI when comparing extreme tertile intakes.	[24]
	Cross-sectional study	1,111 subjects with the average age of 52	IMT-CCA; Presence of focal plaque	No association was found between β-carotene intake and carotid artery IMT or presence of focal carotid artery plaque.	[26]
	Case–control study	Cases: 760 patients with non-fatal AMI; Control: 682 patients	AMI	No significant decrease was found in the adjusted odds of AMI with increasing intake of β-carotene when comparing the highest and the lowest quartile intakes.	[22]
	Prospective cohort study	1,556 employed middle-aged males	Coronary disease mortality	An increase of 3 mg intake of β-carotene was not associated with a decrease in risk of coronary disease mortality.	[27]
Lycopene	Prospective cohort study	73,286 female nurses aged 30–55	CAD incidences	No association was found between the highest quintile of lycopene intake and the CAD risk.	[15]
	Prospective cohort study	Not reported	CHD; CVDs; Stroke	An inverse association was found between a 2.7-fold difference in lycopene intake and the CVD risk for the 5th, 6th and 7th examination average, but not for CHD and stroke risk. An inverse association was found between a 2.7-fold difference in lycopene intake and the CHD risk for 5th and 6th examination average, but not for CVD risk and stroke risk.	[16]
	Prospective cohort study	26,593 Finnish male smokers aged 50–69	Stroke incidences	An inverse association was found between lycopene intake and the risk of cerebral infarction and intracerebral hemorrhage, but no association was significant after simultaneous modeling for other antioxidants.	[14]
	Case–control study	Cases: 760 patients with non-fatal AMI; Control: 682 patients	AMI	No association was found in the adjusted odds of AMI with increasing lycopene intake when comparing the highest and the lowest quartile intakes.	[22]
	Prospective cohort study	39,876 middle-aged and older females	Total CVDs; Vascular events; MI; Stroke	No association was found between lycopene intake and the multivariate-adjusted risk of total CVDs, important vascular event, MI and stroke compared to the lowest quintile.	[17]
Carotenoids with provitamin A activity	Prospective cohort study	5,133 Finnish subjects aged 30–69	CHD mortality	No association was found between the intake of carotenoids with provitamin A activity and the risk of CHD mortality when comparing the highest and lowest tertile intakes.	[28]
	Prospective cohort study	34,486 postmenopausal females	CHD mortality	No association was found between the risk of CHD mortality and the intake of carotenoids with provitamin A activity when comparing the extreme quintile intakes.	[29]
	Case–control study	Cases: 760 patients with non-fatal AMI; Control: 682 patients	AMI	No significant decrease was found in the adjusted odds of AMI when comparing the highest and the lowest quartile intakes.	[22]
	Cross-sectional study	12,773 subjects with the average age of 54	Carotid artery wall thickness	No association was found between the intake of carotenoids with provitamin A activity and the odds for carotid artery plaque when comparing extreme quintile intakes.	[30]
Total carotenoids	Prospective cohort study	725 elderly subjects	Mortality from heart disease	No association was found between total carotenoid intake and the heart disease mortality when comparing the extreme quintile intakes.	[31]
	Case–control study	Cases: 760 patients with non-fatal AMI; Control: 682 patients	AMI	No association was found in the odds of AMI with increasing total carotenoid intake when comparing the highest and the lowest quartile intakes.	[22]

Abbreviations: CAD, coronary artery disease; CVD, cardiovascular disease; CHD, coronary heart disease; AMI, acute myocardial infarction; MI, myocardial infarction; IMT, intima–media thickness; IMT-CCA, intima–media thickness of the common carotid artery; BMI, body mass index.

### 2.4. Lycopene

Jacques et al. [16] published their findings in a prospective study investigating the relationship between dietary lycopene intake and incidences of CVDs, CHD and stroke. The authors analyzed the data from the Framingham Offspring Study cohort, which were derived from the 5th, 6th and 7th study examinations, lasting from year 1991 to 2001, with follow-up for CVD incidences in year 2008. The authors reported a significant inverse association between a 2.7-fold difference in lycopene intake and the CVD risk for the 5th, 6th and 7th examinations in a multivariate model adjusted for age, sex, various CVD risk factors and antioxidant intake (hazard ratio (HR): 0.83, 95% CI: 0.70–0.98). However, no such association was reported for CHD (HR: 0.84, 95% CI: 0.67–1.03) and stroke risk (HR: 0.82, 95% CI: 0.59–1.16). Using data from the 5th and 6th examinations, the authors also found a significant inverse association between a 2.7-fold difference in the lycopene intake and the CHD risk in a multivariate model adjusted for age, sex, various CVD risk factors and antioxidant intake (HR: 0.74, 95% CI: 0.58–0.94), but not for CVDs (HR: 0.86, 95% CI 0.71–1.05) and stroke (HR: 1.07, 95% CI: 0.74–1.54). Similarly, Hirvonen et al. [14] reported a significant inverse association between the lycopene intake and the risk of cerebral infarction (RR: 0.74, 95% CI: 0.59–0.92) and intracerebral hemorrhage (RR: 0.45, 95% CI: 0.24–0.86) but not for subarachnoid hemorrhage (RR: 0.63, 95% CI: 0.33–1.20) after adjusting for age, supplementation group and CVD risk factors. Meanwhile, after simultaneous modeling for other antioxidants, no significant association was found between the lycopene intake and the risks of all forms of stroke. 

However, contradictory evidence was reported by Osganian et al. [15], as the authors highlighted that there was no association between the highest quintile of lycopene intake and the risk of CAD after adjusting for age, smoking status and other CAD risk factors (RR: 0.93, 95% CI: 0.77–1.14). Similarly, Sesso et al. [17] conducted a prospective cohort study involving 29,876 middle-aged and older females who were participating in the Women’s Health Study. During the 7.2-year follow-up, there were 719 cases of CVDs. The authors concluded that there was no association between the highest quintile of lycopene intake and all CVD incidences as compared to the lowest quintile intake after adjusting for age, randomized aspirin, vitamin E and β-carotene treatments and other CVD risk factors. Additionally, in the Milan non-fatal AMI case–control study, Tavani et al. [22] reported no association in the odds of AMI with increasing lycopene intake when comparing the highest and the lowest quartile intakes of lycopene (OR: 1.10, 95% CI: 0.82–1.70) in an unconditional multiple logistic regression model adjusted for age, sex, study, education and other risk factors. 

### 2.5. Carotenoids with Provitamin A Activity

Certain carotenoids such as α-carotene, β-carotene and β-cryptoxanthin exhibit provitamin A activity. In year 1994, Knekt et al. [28] investigated the relationship between the dietary intake of carotenoids with provitamin A activity and CHD mortality in a prospective cohort study. They defined the intake of provitamin A carotenoids as the sum of the intake of β-carotene and half the intake of α-carotene and β-cryptoxanthin. In total, 5,133 Finnish males and females aged 30–69 initially free from CHD were followed-up for 14 years. When comparing the highest and lowest tertiles of provitamin A carotenoid intake, the authors found no significant association between provitamin A carotenoid intake and the risk of CHD mortality for both males (RR: 1.02, 95% CI: 0.70–1.48) and females (RR: 0.62, 95% CI: 0.30–1.29) after adjusting for various risk factors such as age, smoking status and hypertension. Similarly, Kushi et al. [29] prospectively examined the association between the dietary intake of carotenoids with provitamin A activity and the CHD mortality among 34,486 postmenopausal females with no pre-existing CVDs from the Iowa Women’s Health Study cohort. Comparing the extreme quintile of provitamin A carotenoid intake, the authors reported that there was no significant association between a higher intake of carotenoids with provitamin A activity and a decreased risk of CHD mortality (RR: 1.03, 95% CI: 0.63–1.70) after adjusting for age and other predictors of CHD mortality or confounding factors associated with dietary antioxidant intake. Moreover, Tavani et al. [22] concluded that there was no association between the dietary intake of provitamin A carotenoids (sum of the intake of β-carotene and half of the intake of α-carotene, α-and β-cryptoxanthin) and the odds of AMI when comparing the highest and lowest quartile intakes in an unconditional multiple logistic regression model including various confounding variables (OR: 0.76, 95% CI: 0.53–1.09).

Kritchevsky et al. [30] studied the relationship between the intake of carotenoids with provitamin A activity and the presence of carotid artery plaque in 12,773 participants aged 45–64 from the Atherosclerosis Risk in Communities Study. The authors concluded that the inverse association between carotenoid intake and the odds of carotid artery plaque was not significant when adjusting for ethnicity, education, age and various CVD risk for both males (OR: 0.92, 95% CI: 0.75–1.12) and females (OR: 0.84, 95% CI: 0.69–1.04) when comparing the extreme quintiles of intake. Likewise, for non-smokers and former smokers of both sexes, there was no significant association between provitamin A carotenoid intake and the adjusted OR for the prevalence of carotid artery plaque. However, among females who were current smokers, the authors noted a significant decrease in the OR of carotid artery plaques for the highest quintile of provitamin A carotenoid intake as compared to the lowest quintile of provitamin A intake in females who were currently smoking (OR: 0.67, 95% CI: 0.45–0.98). 

### 2.6. Total Carotenoids

In year 1996, Sahyoun et al. [31] published their findings from the Nutrition Status Survey cohort study by investigating the association between the mortality from heart diseases and the total carotenoid intake. In this study, total carotenoids included lutein plus zeaxanthin, α-carotene, β-carotene, β-cryptoxanthin and lycopene. No significant inverse association was observed between the total carotenoid intake and the heart disease mortality when compared the extreme quintiles of carotenoid intake (RR: 0.80, 95% CI: 0.50–1.26) in the Cox proportional hazard model adjusted for age, sex, serum cholesterol, disease status, physical ailments affecting shopping and vitamin C and E intakes. This finding was corroborated by the results of the Milan non-fatal AMI case–control study conducted by Tavani et al. [22], who defined total carotenoids as the sum of lutein plus zeaxanthin, α-carotene, β-carotene, γ-carotene, ζ-carotene, α-cryptoxanthin, β-cryptoxanthin and lycopene. The authors also noted no association between the OR of AMI and total carotenoid intake when comparing the highest and lowest quartiles of intake (OR: 0.84, 95% CI: 0.58–1.21) in an unconditional multiple logistic regression model including various confounding variables such as age, sex, study, education and CVD risk factors. 

### 2.7. Conflicting Reporting Results

Conflicting results exist in the association between the dietary intake of carotenoids and the risk of CVDs, and there are several possible explanations for this discrepancy. Firstly, the populations among the different studies were heterogeneous. For instance, Hirvonen et al. [14] published their findings based on male smokers, who may be at a greater exposure to the oxidative stress. However, such findings may not apply to non-smokers. Moreover, the conflicting results are not particularly surprising in light of the fact that the observational studies have different study designs. Other potential reasons for contradictory evidence may include different adjustment factors used in multivariate analyses, the different methods used in statistical analyses and the different time frames in the prospective studies. 

## 3. Randomized Controlled Trials on Carotenoids and CVDs

**Scheme 1:**
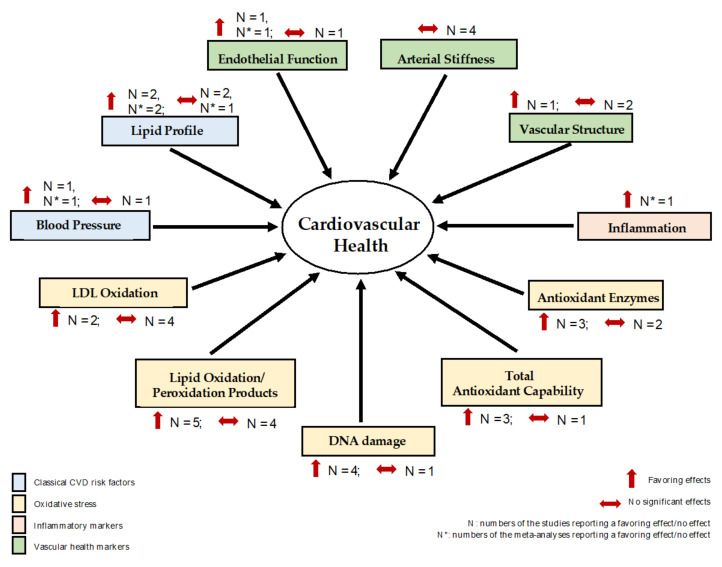
Effects of dietary intake of carotenoids on cardiovascular health. Abbreviations: LDL, low-density lipoprotein.

Extensive evidence indicates that carotenoids play important roles in preventing and managing CVDs. Table 2 outlines the results of randomized controlled trials (RCTs) that have investigated the effects of dietary intake of carotenoids on cardiovascular health. The section below summarizes the effects mainly by assessing the classical CVD risk factors and the secondary CVD risk factors, with the effects further illustrated in Scheme 1. The former classical CVD risk factors refer to the lipid–lipoprotein profile and blood pressure. The latter secondary CVD risk factors are generally represented by oxidative stress, inflammatory markers and vascular-health-related parameters. Although the beneficial effects of carotenoids on CVD protection are suggested, the data are controversial overall. 

### 3.1. Classical CVD Risk Factors

The blood lipid–lipoprotein profile, including triglycerides (TG), total cholesterol (TC), high-density lipoprotein cholesterol (HDL-C), LDL cholesterol (LDL-C) and very low-density lipoprotein cholesterol (VLDL-C), as well as blood pressure, including systolic blood pressure (SBP) and diastolic blood pressure (DBP), are well-known classical risk factors for CVDs. 

Epidemiology studies have demonstrated the association between an impaired lipid–lipoprotein profile and the risk of CVDs [32,33,34]. A recently published systematic review and meta-regression reported that the RR for cardiovascular events was 0.91 per 1 mmol/L reduction in TG and was 0.79 per 1 mmol/L reduction in LDL-C [35]. Xu et al. [36] demonstrated that lutein supplementation controlled the lipid–lipoprotein profile in early atherosclerosis patients by significantly reducing 0.7 mmol/L TG and 0.3 mmol/L LDL-C compared to baseline. Goji berry, also known as Lycium barbarum, is rich in carotenoids, especially zeaxanthin. de Souza-Zanchet et al. [37] depicted favorable effects on TC, HDL-C, LDL-C and VLDL-C with 14 g of Goji berry consumption for 45 days in individuals with metabolic syndrome. However, this effect was not observed with lutein supplementation or β-cryptoxanthin-rich juice consumption in healthy participants [38,39]. Moreover, a meta-analysis that compiled the data from six RCTs, including 401 participants, reported that consuming tomato products (70 to 400 g per day) significantly reduced LDL by 0.22 mmol/L, whereas other blood lipids such as TC, HDL-C and TG were not significantly improved [40]. Consistent findings for the changes of blood lipids were revealed by another meta-analysis, which investigated lycopene and lycopene-rich foods (mainly tomato products) [41]. Although lycopene is the main carotenoid in tomato products, a meta-analysis failed to observe a lipid-modulating effect with pure lycopene supplementation. Similarly, another meta-analysis reported no beneficial effect of astaxanthin supplementation on the plasma TG, TC, LDL-C and HDL-C [42].

The Seventh Report from National High Blood Pressure Education Program showed that for every 20 mm Hg increase in SBP or 10 mm Hg increase in DBP, the mortality from ischemic heart disease and stroke doubled [43]. Furthermore, a meta-regression illustrated that each 5 mmHg decrease in SBP and 2 mmHg decrease in DBP was associated with 13% and 12% lower risk of stroke, respectively [44]. The meta-analysis mentioned above revealed a reduction of SBP by 5.6 mmHg with 4 to 30 mg daily lycopene supplementation [40]. A recently published RCT conducted a dose–response study by comparing tomato nutrient complexes containing 5 mg, 15 mg and 30 mg of lycopene [45]. The authors reported that the SBP-lowering effect was observed with the 15 mg and 30 mg lycopene groups but was not with the 5 mg lycopene group. This suggests the possible effect of lycopene on the prevention of CVDs is caused by a substantial SBP reduction, whereas this favoring effect may require a certain dosage of the lycopene supplementation. Interestingly, a favoring effect on blood pressure was not observed with lycopene-rich tomato products [40] or β-cryptoxanthin-rich juice [39]. This suggests the influence of food processing on carotenoid-rich products cannot be overlooked and that the bioavailability of the carotenoid supplement and the carotenoids in their rich sources may be different. 

### 3.2. Oxidative Stress

Recent research has suggested that oxidative stress acts as a trigger of atherosclerosis and that excessive production of ROS is known to induce adverse pathological conditions, which may eventually contribute to the progression of several cardiovascular disorders [46,47]. LDL oxidation [46,47], lipid oxidation or peroxidation products, total antioxidant capability, antioxidant enzyme activity and DNA damage are several established markers that are used to evaluate oxidative stress status.

#### 3.2.1. LDL Oxidation

The oxidation of LDL in the vessels is implicated in the pathogenesis of atherosclerotic lesions and thrombus formation. An elevated plasma-oxidized LDL (ox-LDL) level has been documented to be associated with a higher risk of subclinical atherosclerosis [48], CHD [49], myocardial infarction (MI) [50] and cardiac events [51]. Apart from the direct ox-LDL level, the LDL lag time and LDL oxidation rate are also commonly measured to describe the susceptibility of LDL oxidation. Several RCTs have been conducted to investigate the effects of lycopene on LDL oxidation by assessing the ox-LDL level and the LDL oxidation rate [52,53,54], and none of these studies have observed an attenuated trend in the LDL oxidation. Additionally, a meta-analysis compiling data from three trials reported that lycopene supplementation did not prolong the lag time of LDL [55]. 

In contrast, Kiokias and Gordon [56] found a significant increase in the LDL lag time after consuming carotenoid mixtures comprising carotenes and xanthophylls. This finding was in agreement with the study conducted by Lwamoto et al. [57], who described that astaxanthin intake dose-dependently prolonged the LDL lag time. This suggests that astaxanthin may play a beneficial role in suppressing atherogenicity by inhibiting the initial LDL oxidation. Unlike other carotenoids, astaxanthin contains hydroxy and keto moieties on the terminal ionone ring in addition to a conjugated polyene chain at the center [58]. This unique structure makes it capable of scavenging radicals in both lipophilic and hydrophilic environments, which may explain the reason that a suppressed LDL oxidation effect was only observed with certain types of xanthophylls.

#### 3.2.2. Lipid Oxidation/Peroxidation Products

Malondialdehyde (MDA) is a final product of polyunsaturated fatty acid peroxidation and is one of the most widely measured biomarkers of oxidative stress [59]. Several RCTs have examined the effects of carotenoid supplements on MDA levels [38,54,60,61,62]. Kasperczyk et al. [60,62] conducted a trial with the population who were chronically exposed to lead and found that the oxidative stress biomarkers MDA and lipid hydroperoxides (LHP) both significantly decreased with the oral consumption of 10 mg β-carotene for 12 weeks. Simultaneously, a reduction in MDA was also observed in the 20 mg lutein supplementation group reported by Wang et al. [38]. These findings suggest β-carotene and lutein may actively react with lipid peroxyl radicals, leading to a lower generation of peroxidation products. Two RCTs consistently suggested a beneficial role of zeaxanthin-rich Goji berries in attenuating oxidative stress, as evidenced by decreased levels of the lipid peroxidation products MDA [37] and 8-iso-prostaglandin F2α (8-iso-PGF2α) [63]. 

On the contrary, lycopene supplementation failed to decrease MDA and hydroxyl nonenal (HNE) levels, as reported by two placebo-controlled RCTs in healthy populations [54,61]. The 8-iso-PGF2α level was also unaltered with supplementation of a lycopene-rich tomato-based drink [64]. However, a decreased MDA was evident in postmenopausal females with the lycopene supplementation [65]. Given that postmenopausal females may experience an elevated burden of oxidative stress [66], the effect of lycopene on MDA levels may be more pronounced with females in the postmenopausal state.

#### 3.2.3. Antioxidant Capability

The total antioxidant capability (TAOC) refers to the cumulative effects of antioxidants present in blood and is commonly measured to evaluate the antioxidant response against free radicals [67]. Epidemiological evidence has suggested that a lower TAOC may be associated with a higher incidence of CVDs [68,69,70]. Kiokias et al. [35] reported that the plasma antioxidant capability assessed by the oxygen radical absorbance capacity was not influenced by a mixture of carotenoid supplementation for 3 weeks among the healthy population. In contrast, the serum TAOC increased among individuals with metabolic syndromes after consuming zeaxanthin-rich Goji berries for 45 days [37]. Moreover, studies with a longer intervention duration, such as 12 weeks of lutein supplementation [38] and 4 months of lycopene capsules or lycopene-rich tomato juice consumption [65], both consistently reported increased plasma TAOC. 

#### 3.2.4. Antioxidant Enzymes

Glutathione peroxidase (GPx), catalase (CAT) and superoxide dismutase (SOD) are well-known enzymes involved in the antioxidant response. Several RCTs have measured the activity of these antioxidant enzymes when investigating the effects of carotenoids or carotenoid-rich foods against oxidative stress. 

Kasperczyk et al. [60,62] reported that the activities of SOD and its isoenzyme extracellular SOD, CAT and glucose-6-phosphate dehydrogenase (G6PD) were higher in the β-carotene supplementation group. However, the activity of GPx decreased and the activities of glutathione reductase and glutathione-S-transferase were unchanged. The activity of plasma SOD also increased with lycopene supplementation in the male population as revealed by a placebo-controlled randomized trial [71]. In addition, increased CAT activity and decreased SOD activity were reported upon consumption of zeaxanthin-rich Goji berry for 45 days with metabolic syndrome patients [37]. In contrast, 12 weeks of lutein supplementation [38] or 4 months of lycopene capsules or lycopene-rich tomato juice consumption [65] did not alter the activity of any antioxidant enzymes including SOD, GPx and CAT in healthy participants or postmenopausal females. Undeniably, discrepancies in the activity levels of antioxidant enzymes existed in the response to carotenoid consumption, and differences were even observed within the same type of enzyme. At present, evidence regarding the effects of carotenoids on these enzymes is still insufficient to draw a definite conclusion. Future investigations are required to elucidate the effects of carotenoids on the activity levels of antioxidant enzymes.

#### 3.2.5. DNA Damage

Deoxyribonucleic acid (DNA) damage is one of the major consequences of oxidative stress, whereby the generation of ROS exceeds the antioxidant defense capacity of the cell. One of the most widely used methods to quantitatively assess DNA damage is the comet assay, in which the DNA tail length, tail intensity and tail moment are commonly measured [72]. In addition, 8-hydroxy-2-deoxyguanosine (8-OHdG), a predominant form of free-radical-induced lesions of DNA, is also frequently used to indicate the extent of oxidative damage [73]. 

Two previous RCTs from Zhao et al. [74] and Torbergsen and Collins [75] assessed three types of carotenoids, namely lutein, β-carotene and lycopene, and these two studies reported consistent findings. Zhao et al. [74] conducted a relatively longer intervention whereby subjects consumed lutein, β-carotene, lycopene or a mixture of these three carotenoids for 56 days. The authors revealed that all types of carotenoid supplementation significantly decreased DNA damage, as evidenced by a shorter DNA tail length. Furthermore, β-carotene seemed to have a more pronounced beneficial effect as significantly less DNA damage was found with β-carotene as early as day 15. In agreement with Zhao et al., Torbergsen and Collins [75] also observed a decrease in DNA tail intensity in the β-carotene supplementation group. However, they did not see any changes in DNA tail intensity for the one-week supplementation of lutein. It is plausible that lutein may require a longer period to exert a substantial favorable effect on DNA damage. Moreover, a combination of carotenoids (lutein, β-carotene and lycopene) was also reported to lower DNA damage, as evidenced by decreased DNA degradation products (as assessed by the 8-OHdG/creatinine ratio in urine) [56] and a reduced DNA tail length [74]. 

A meta-analysis reported a decreased DNA tail length in the lycopene supplementation group, suggesting a favorable effect against DNA damage [55]. However, the level of 8-OHdG was unchanged after consuming lycopene for 3 weeks, as reported by a RCT [61]. Simultaneously, Torbergsen and Collins [75] observed a decreased DNA tail intensity with lycopene, while the decrease was only significant with the participants whose plasma lycopene concentrations were elevated after lycopene intervention. This was presumably due to the lower bioavailability of lycopene as compared to other carotenoids and because the potential protection of lycopene against DNA damage by oral administration may be masked by its poor bioavailability.

### 3.3. Inflammatory Biomarkers

Inflammation plays a pivotal role in the initiation, progression and manifestation of CVDs [6]. Extensive literature has demonstrated that the elevated blood pro-inflammatory cytokines such as C-reactive protein (CRP), interleukin-6 (IL-6) and tumor necrosis factor-α (TNF-α) are associated with CVD events [76,77,78,79]. A very recently published systematic review compiled data from RCTs and further performed a meta-analysis [80], finding that overall carotenoid supplementation significantly reduced CRP and IL-6 levels, whereas the effect on TNF-α was not substantial. However, it is worth noting that the decrease in the levels of TNF-α became statistically significant after removing one RCT, whereby subjects had a relatively lower level of TNF-α at baseline. The authors inferred that the effectiveness of carotenoids on these biomarkers might be prone to occur with higher levels of pro-inflammatory cytokines at baseline [80]. 

For the individual carotenoids, this protective effect on inflammatory biomarkers seemed to be less potent and the results were mixed. Lutein or zeaxanthin, astaxanthin and β-cryptoxanthin showed to reduce CRP levels, β-carotene and crocin showed no effect on CRP levels, and lycopene was the only carotenoid that caused a reduction in IL-6 levels, although it did not affect the CRP level [80]. These mixed findings suggest that the protective effect against the inflammatory biomarkers is dependent on the individual type of carotenoid. Additionally, subgroup analysis revealed that higher than 10 mg lutein decreased the CRP level while less than 10 mg lutein did not, suggesting that a certain amount of carotenoids might be required to exert an anti-inflammatory effect.

### 3.4. Vasular Health

#### 3.4.1. Endothelial Function

The vascular endothelium, a delicate monolayer of cells lining the interior surface of the entire cardiovascular system, exerts a substantial role in vascular function. A properly functioning endothelium is considered one of the best indicators of vascular health, whereas endothelial dysfunction may lead to CVD progression [81,82]. Several measurements can be used to assess the endothelial function, with brachial artery flow-mediated dilation (FMD) and reactive hyperemia–peripheral arterial tonometry (RH-PAT) being the two most rapidly used methods in clinical research [83]. 

A 1% increase of FMD was reported to be associated with a reduction of 10% RR of CVD events and all-cause mortality [84]. A recent meta-analysis evaluated the effect of consuming lycopene-rich tomato products on FMD by compiling data from 6 RCTs, including 233 participants [40], whereby tomato consumption significantly improved FMD by 2.53%. However, this beneficial effect was only observed with long-term tomato consumption (at least one week) but not with acute feeding. In addition, no favorable effect on FMD was found from consuming Goji berry, which is rich in zeaxanthin [85]. 

Although RH-PAT is a relatively new test compared to FMD, its usage has been rapidly increasing over the past two decades [86]. It was revealed that per 0.1 increase in the RH-PAT index was associated with 15% lower rate of cardiovascular events [84]. In line with the findings of the above meta-analysis, Kim et al. [71] also found a beneficial effect on endothelial function with 15 mg lycopene supplementation per day for 8 weeks, as evidenced by a 0.34 increase in the RH-PAT index. 

#### 3.4.2. Arterial Stiffness

Arterial stiffness indicates the rigidity of the arterial wall and is regarded as a vital marker for vascular health, because it affects the pressure, blood flow and changes in arterial diameter with each heartbeat [87]. Arterial stiffness can be measured by pulse wave velocity (PWV) and augmentation index (AI) [88]. Increased arterial stiffness has often been associated with a higher risk of CVD events [89,90].

A previous RCT reported no differences in PWV and AI levels between a lycopene-treated group and placebo group, whether for healthy volunteers or CVD patients [52]. Similarly, another study also failed to observe an improvement in arterial stiffness even with a relatively higher daily consumption of tomato-based products (equivalent to 32–50 mg lycopene) [53]. Additionally, Nakamura et al. [39] compared the effects of 12 weeks consumption of β-cryptoxanthin-rich vs. β-cryptoxanthin-deprived satsuma mandarin juice on PWV, finding no significant differences between these two groups. Moreover, 12 months of astaxanthin supplementation also did not result in an improvement in arterial stiffness, as evidenced by the values of PWV and AI [91]. Although caution in interpretation is required, the present evidence does not support the mechanism of CVD protection effects from carotenoids by attenuating arterial stiffness.

#### 3.4.3. Vascular Structure

Carotid artery IMT, a marker of vascular structure, is considered an early subclinical measure of atherosclerosis and is commonly accepted to predict CVD events [92]. It was reported that with each 0.1 mm difference in IMT, the risks of MI and stroke increased by up to 15% and 18%, respectively [93]. 

Zou et al. [94] demonstrated that 12 months of lutein supplementation significantly decreased IMT by 0.035 mm among subclinical atherosclerosis populations. A more pronounced reduction in IMT by 0.073 mm was observed when consuming lutein and lycopene together. On the contrary, 12 months of astaxanthin supplementation failed to result in a significant improvement in IMT with healthy participants [91]. Moreover, Toh et al. [85] reported no significant reduction in IMT after consuming Goji berry for 16 weeks in relatively healthy middle-aged and older adults. This was possibly because subjects who were diagnosed with subclinical atherosclerosis had an elevated IMT at baseline compared with healthy subjects. Thus, carotenoids may play a more effective role in attenuating IMT with participants who already have an impaired vascular structure.

**Table 2:** Evidence of the effects of carotenoids on CVD health from randomized controlled trials.

Carotenoids	Study Design	Intervention	Outcome	References
Classical CVD Risk Factors				
Lutein	RCT, parallel, double-blinded, Healthy subjects (*n* = 117)	10, 20 mg lutein for 12 weeks	↔ TG, LDL-C, HDL-C	[38]
	RCT, parallel, double-blinded, Early atherosclerosis patients (*n* = 65)	20 mg lutein for 12 weeks	↓ TG, LDL-C ↔HDL	[36]
Zeaxanthin	RCT, parallel, Metabolic syndrome patients (*n* = 50)	14 g Goji berry for 45 days	↓ TC, LDL-C, VLDL-C ↑ HDL-C	[37]
β-cryptoxanthin	RCT, parallel, double-blinded, Healthy subjects (*n* = 117)	β-cryptoxanthin-rich satsuma mandarin juice for 12 weeks	↔ LDL-C, HDL-C, TG, SBP, DBP	[39]
Astaxanthin	Meta-analysis	Astaxanthin	↔ TC, LDL-C, HDL-C, TG	[42]
Lycopene	RCT, crossover, double-blinded, Hypertensive subjects (*n* = 46)	Tomato nutrient complex (equivalent to 5, 15 and 30 mg lycopene) for 8 weeks	↓ SBP	[45]
	Meta-analysis	Lycopene or lycopene-rich foods	↓ LDL-C ↔ TC, HDL-C, TG	[41]
	Meta-analysis	Lycopene	↓ SBP ↔ DBP, TC, LDL-C, HDL-C, TG	[40]
	Meta-analysis	Tomato	↓ LDL-C, ↔ SBP, DBP, TC, HDL-C, TG	[40]
**Oxidative Stress**				
LDL oxidation				
Astaxanthin	RCT, parallel, open labeled, Healthy subjects (*n* = 24)	1.8, 3.6, 14.4, 21.6 mg astaxanthin for 2 weeks	↑ LDL lag-time	[57]
Lycopene	RCT, parallel, double-blinded, CVD patients (*n* = 36), Healthy subjects (*n* = 36)	7 mg Lycopene for 2 months	↔ ox-LDL	[52]
	RCT, parallel, single-blinded, Healthy subjects (*n* = 225)	Tomato or 10 mg lycopene capsules for 12 weeks	↔ ox-LDL	[53]
	RCT, parallel, double-blinded, Healthy subjects (*n* = 77)	6.5, 15, 30 mg lycopene for 8 weeks	↔ LDL oxidation rate	[54]
	Meta-analysis	Lycopene	↔ LDL lag-time	[55]
Carotenoid mixture	RCT, crossover, double-blinded, Healthy subjects (*n* = 31)	4.4 mg lutein, 6.0 mg β-carotene, 1.4 mg α-carotene, 4.5 mg lycopene, 11.7 mg bixin and 2.2 mg paprika for 3 weeks	↑ LDL lag-time	[56]
Lipid oxidation/peroxidation products				
Lutein	RCT, parallel, double-blinded, Healthy subjects (*n* = 117)	10, 20 mg lutein for 12 weeks	↓ MDA	[38]
Zeaxanthin	RCT, parallel, Metabolic syndrome patients (*n* = 50)	14 g Goji berry for 45 days	↓ MDA	[37]
	RCT, parallel, double-blinded, Healthy subjects (*n* = 40)	15 g Goji berry for 16 weeks	↓ 8-iso-PGF2α, ↔ MDA	[63]
Astaxanthin	RCT, parallel, double-blinded, Healthy subjects (*n* = 58)	12 mg astaxanthin for 12 months	↔ F2-isoPs	[91]
β-carotene	RCT, parallel, Healthy male workers exposed to lead (*n* = 82)	10 mg β-carotene for 12 weeks	↓ MDA, LHP	[60,62]
Lycopene	RCT, parallel, double-blinded, Healthy subjects (*n* = 77)	6.5, 15, 30 mg lycopene for 8 weeks	↔ MDA, HNE	[54]
	RCT, parallel, double-blinded, Healthy males (*n* = 105)	30 mg lycopene for 3 weeks	↔ MDA	[61]
	RCT, parallel, Postmenopausal females (*n* = 60)	Regular, lycopene-rich tomato juice (equivalent to 30 and 70 mg lycopene), 30 mg lycopene capsules for 6 months	↓ MDA	[65]
	RCT, crossover, double-blinded, Healthy subjects (*n* = 26)	Tomato-based drink for 26 days	↔ 8-iso-PGF2α	[64]
Total antioxidant capability				
Lutein	RCT, parallel, double-blinded, Healthy subjects (*n* = 117)	10, 20 mg lutein for 12 weeks	↑ TAOC	[38]
Zeaxanthin	RCT, parallel, Metabolic syndrome patients (*n* = 50)	14 g Goji berry for 45 days	↑ TAOC	[37]
Lycopene	RCT, parallel, Postmenopausal females (*n* = 60)	Regular juice, lycopene-rich tomato juice (equivalent to 30 and 70 mg lycopene), 30 mg lycopene capsules for 6 months	↑ TAOC	[65]
Carotenoid mixture	RCT, crossover, double-blinded, Healthy subjects (*n* = 31)	4.4 mg lutein, 6.0 mg β-carotene, 1.4 mg α-carotene, 4.5 mg lycopene, 11.7 mg bixin and 2.2 mg paprika for 3 weeks	↔ ORAC	[56]
Antioxidant enzymes				
Lutein	RCT, parallel, double-blinded, Healthy subjects (*n* = 117)	10, 20 mg lutein for 12 weeks	↔ SOD, GPx, CAT	[38]
Zeaxanthin	RCT, parallel, Metabolic syndrome patients (*n* = 50)	14 g Goji berry for 45 days	↓ SOD ↑ CAT	[37]
β-carotene	RCT, parallel, Healthy male workers exposed to lead (*n* = 82)	10 mg β-carotene for 12 weeks	↑ SOD, EC-SOD, CAT, G6PD ↓ GPx ↔ GR, GST	[60,62]
Lycopene	RCT, parallel, Postmenopausal females (*n* = 60)	Regular, lycopene-rich tomato juice (equivalent to 30 and 70 mg lycopene), 30 mg lycopene capsules for 6 months	↔ SOD, GPx, CAT	[65]
	RCT, parallel, double-blinded, Healthy males (*n* = 126)	6, 15 mg lycopene for 8 weeks	↑ SOD	[71]
DNA damage				
Lutein	RCT, crossover, Healthy subjects (*n* = 8)	15 mg lutein for 1 week	↔ DNA tail intensity	[75]
	RCT, parallel, double-blinded, Postmenopausal females (*n* = 37)	12 mg lutein for 56 days	↓ DNA tail length	[74]
β-carotene	RCT, parallel, double-blinded, Postmenopausal females (*n* = 37)	12 mg β-carotene for 56 days	↓ DNA tail length	[74]
	RCT, crossover, Healthy subjects (*n* = 8)	15 mg β-carotene for 1 week	↓ DNA tail intensity	[75]
Lycopene	RCT, parallel, double-blinded, Postmenopausal females (*n* = 37)	12 mg lycopene for 56 days	↓ DNA tail length	[74]
	RCT, crossover, Healthy subjects (*n* = 8)	15 mg lycopene for 1 week	↓ DNA tail intensity	[75]
	RCT, parallel, double-blinded, Healthy males (*n* = 105)	30 mg lycopene for 3 weeks	↔ 8-OHdG	[61]
	Meta-analysis	Lycopene	↓ DNA tail length	[55]
Carotenoid mixture	RCT, parallel, double-blinded, Postmenopausal females (*n* = 37)	4 mg lutein, 4 mg β-carotene and 4 mg lycopene for 56 days	↓ DNA tail length	[74]
	RCT, crossover, double-blinded, Healthy subjects (*n* = 31)	4.4 mg lutein, 6.0 mg β-carotene, 1.4 mg α-carotene, 4.5 mg lycopene, 11.7 mg bixin and 2.2 mg paprika for 3 weeks	↓ 8-OHdG: creatinine	[56]
**Inflammatory Markers**				
Lutein/zeaxanthin	Meta-analysis	Lutein/zeaxanthin	↓ CRP ↔ IL-6, TNF-α	[80]
Astaxanthin	Meta-analysis	Astaxanthin	↓ CRP ↔ IL-6, TNF-α	[80]
β-cryptoxanthin	Meta-analysis	β-cryptoxanthin	↓ CRP ↔ IL-6, TNF-α	[80]
β-carotene	Meta-analysis	β-carotene	↔ CRP, IL-6	[80]
Lycopene	Meta-analysis	Lycopene	↓ IL-6 ↔ CRP, TNF-α	[80]
Crocin	Meta-analysis	Crocin	↔ CRP, IL-6, TNF-α	[80]
Overall carotenoids	Meta-analysis	Overall carotenoids	↓ CRP, IL-6 ↔ TNF-α	[80]
**Vascular Health Markers**				
Endothelial function				
Zeaxanthin	RCT, parallel, double-blinded, Healthy subjects (*n* = 40)	15 g Goji berry for 16 weeks	↔ FMD	[85]
Lycopene	Meta-analysis	Tomato	↑ FMD	[40]
	RCT, parallel, double-blinded, Healthy males (*n* = 126)	6, 15 mg lycopene for 8 weeks	↑ RH-PAT	[71]
Arterial stiffness				
β-cryptoxanthin	RCT, parallel, double-blinded, Healthy subjects (*n* = 117)	β-cryptoxanthin–rich satsuma mandarin juice for 12 weeks	↔ PWV	[39]
Astaxanthin	RCT, parallel, double-blinded, Healthy subjects (*n* = 58)	12 mg astaxanthin for 12 months	↔ PWV, AI	[91]
Lycopene	RCT, parallel, double-blinded, CVD patients (*n* = 36), Healthy subjects (*n* = 36)	7 mg lycopene for 2 months	↔ PWV, AI	[52]
	RCT, parallel, single-blinded, Healthy subjects (*n* = 225)	Tomato or 10 mg lycopene capsules for 12 weeks	↔ PWV	[53]
Vascular structure				
Lutein	RCT, parallel, double-blinded, Subjects with subclinical atherosclerosis (*n* = 144)	20 mg lutein for 12 months	↓ IMT	[94]
Zeaxanthin	RCT, parallel, double-blinded, Healthy subjects (*n* = 40)	15 g Goji berry for 16 weeks	↔ IMT	[85]
Astaxanthin	RCT, parallel, double-blinded, Healthy subjects (*n* = 58)	12 mg astaxanthin for 12 months	↔ IMT	[91]
Carotenoid mixture	RCT, parallel, double-blinded, Subjects with subclinical atherosclerosis (*n* = 144)	20 mg lutein and 20 mg lycopene for 12 months	↓ IMT	[94]

Abbreviations: RCT, randomized controlled trial; TG, triglycerides; LDL-C, low-density lipoprotein cholesterol; HDL-C, high-density lipoprotein cholesterol; TC, total cholesterol; VLDL-C, very low-density lipoprotein cholesterol; SBP, systolic blood pressure; DBP, diastolic blood pressure; ox-LDL, oxidized low-density lipoprotein; LDL, low-density lipoprotein; MDA, malondialdehyde; 8-iso-PGF2α, 8-iso-prostaglandin F2α; LHP, lipid hydroperoxides; HNE, hydroxyl nonenal; F2-isoPs, F2-isoprostanes; ORAC, oxygen radical absorbance capacity; TAOC, total antioxidant capacity; SOD, superoxide dismutase; EC-SOD, extracellular superoxide dismutase; CAT, catalase; G6PD, glucose-6 phosphate dehydrogenase; GPx, glutathione peroxidase; GR, glutathione reductase; GST, glutathione-S-transferase; DNA, deoxyribonucleic acid; 8-OHdG, 8-hydroxy-2′-deoxyguanosine; CRP, C-reactive protein; IL-6, interleukin-6; TNF-α, tumor necrosis factor-α; FMD, flow-mediated dilatation; RH-PAT, reactive hyperemia–peripheral arterial tonometry; PWV, pulse wave velocity; AI, augmentation index; IMT, carotid artery intima–media thickness.

### 3.5. Conflicting Reporting Results

There is no denying that the effects of carotenoid intake on the classical or secondary CVD risk factors reported by intervention studies are not always consistent, and there are several possible explanations for this discrepancy. Firstly, the populations studied among different RCTs are heterogeneous. For instance, the beneficial effects of carotenoid intake seem to be more pronounced with the populations who have metabolic syndrome, experience greater oxidative stress or inflammation or with an impaired vascular structure than healthy participants. Secondly, in light of the differences in physicochemical properties of carotenoids, the protective effects against certain CVD biomarkers may vary. Thirdly, considering that other antioxidant nutrients (e.g., vitamin C) that are contained in natural foods may also play a role in the CVD markers, the mixed findings can be attributed to the intake of pure carotenoid supplements or consumption of the carotenoid-rich foods. Moreover, the dosage of carotenoid intake and the length of the intervention period could also result in a discrepancy.

## 4. Association between Circulating or Adipose Carotenoid Levels and CVDs

Although the results regarding the association between carotenoid consumption and the risk for various CVDs remain controversial, as discussed above, several epidemiological studies that investigated the association between the circulating carotenoid concentrations and CVDs have published relatively consistent findings. A significant inverse relationship between the serum α-carotene levels and CVD mortality was supported by two large cohort studies [95,96]. Moreover, many studies consistently reported that the circulating or adipose carotenoid levels were inversely related to CVD events [18,19,97,98,99]. The specifics of these observational studies that reported an inverse association between the carotenoid concentrations in blood or adipose tissue and the risk of CVDs are present in Table 3. Although a systematic approach to literature review is required for valid interpretation, the evidence that supports an association between the dietary carotenoid intake and CVD protection seem to be weaker as compared with the circulating carotenoid levels.

**Table 3:** Inverse associations between the circulating or adipose carotenoids and the risk of CVDs.

Carotenoids	Study Design	Subjects	Outcome	Main Results	References
α-carotene	Nested case–control study	Cases: 297 males with ischemic stroke; Control: 297 paired males	Ischemic stroke	An inverse association from the second to fifth quintile was found between the plasma α-carotene concentrations and the risk of ischemic stroke.	[18]
	Prospective cohort study	5133 Japanese subjects aged 39–80	CVD mortality	Higher serum concentrations of α-carotene were associated with lower risk of CVDs.	[95]
	Prospective cohort study	13,293 US subjects	CVD mortality	Higher serum concentrations of α-carotene were associated with lower risk of CVDs.	[96]
β-carotene	Prospective cohort study	5133 Japanese subjects aged 39–80	CVD mortality	Higher serum concentrations of β-carotene were associated with lower risk of CVDs.	[95]
	Nested case–control study	Cases: 123 subjects with MI; Control: 123 paired subjects	MI	An inverse association was found between the serum β-carotene concentrations and the risk of MI.	[98]
	Case–control study	Cases: 662 subjects with acute MI; Control: 717 subjects	Acute MI	An inverse association was found between the concentrations of β-carotene in adipose tissue and the risk of acute MI when comparing the highest and the lowest quintiles.	[99]
Carotenes	Prospective cohort study	2974 Basel working males	IHD mortality	Lower plasma concentrations of the carotenes were associated with higher risk of IHD.	[19]
Lycopene	Prospective cohort study	5133 Japanese subjects aged 39–80	CVD mortality	Higher serum concentrations of lycopene were associated with lower risk of CVDs.	[95]
	Case–control study	Cases: 662 subjects with acute MI; Control: 717 subjects	Acute MI	Higher concentrations of lycopene in adipose tissue was associated with lower risk of acute MI.	[99]
Carotenoids	Prospective cohort study	1899 males with hyperlipidemia, aged 40–59	CHD	An inverse association was found between the serum carotenoid concentrations and the risk of CHD when comparing the highest and the lowest quartiles.	[97]

Abbreviations: IHD, ischemic heart disease; CVD, cardiovascular disease; CHD, coronary heart disease; CAD, coronary artery disease; MI, myocardial infarction.

Moreover, as mentioned in the earlier section, Torbergsen and Collins [75] failed to observe the prevention effects of lycopene intake on DNA damage. However, when they evaluated the DNA damage only with the subjects whose plasma lycopene concentrations were elevated after the lycopene intervention, a significant decrease in DNA tail intensity became evident. This suggests that a desired bioavailability of carotenoids must be ensured in order to obtain their health-promoting effects.

Furthermore, the discrepancies in CVD protection effects between carotenoid intake and circulating carotenoid levels were identified by meta-analysis. Aune et al. [100] compiled all data from the prospective cohort and nested case–control studies, which reported the association between carotenoids from diet or blood concentrations and the RR of CHD, stroke and CVDs. They found that the intake of β-carotene was associated with 8–19% reductions in the RR of CHDs and stroke, but there was no association for CVDs. However, a 15–24% decrease in RR was evident for blood β-carotene levels in relation to all CHD, stroke and CVDs. Additionally, a nonlinear association between blood lycopene concentrations and stroke was observed, while this nonlinear association was not significant with the lycopene intake. Taken together, these results underline the importance of bioavailability, which must be considered when evaluating the roles of carotenoids in cardiovascular health.

## 5. Dietary Strategies for the Improvement of Carotenoid Bioavailability

**Scheme 2:**
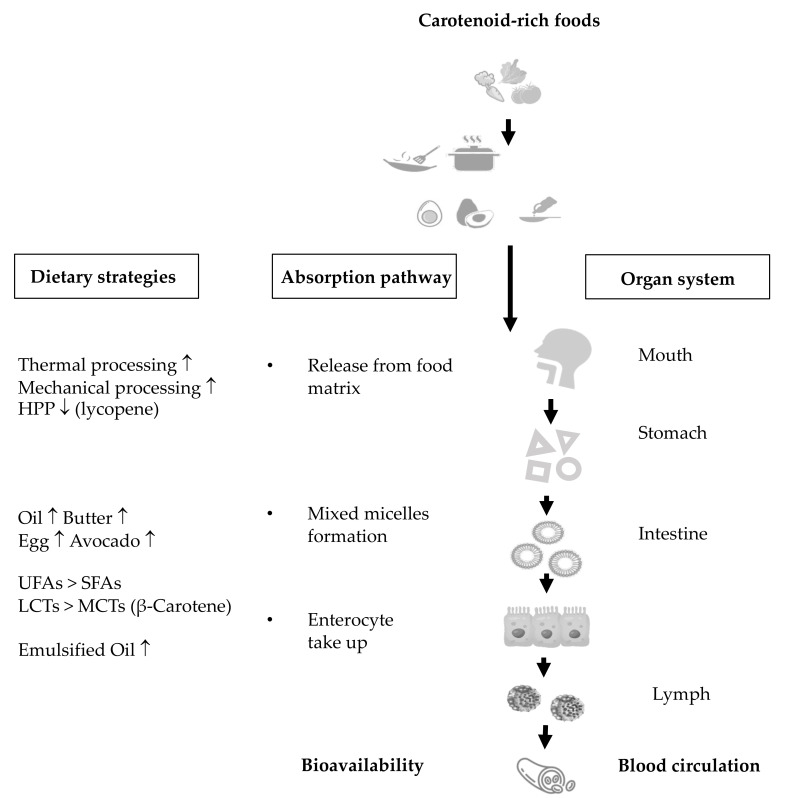
Dietary strategies for improving carotenoid bioavailability. Abbreviations: HPP, high-pressure homogenization processing; SFAs, saturated fatty acids; UFAs, unsaturated fatty acids; LCTs, long-chain triglycerides; MCTs, medium-chain triglycerides.

Carotenoid bioavailability refers to the proportion of carotenoids or their metabolites that are available for usage or storage upon consumption [101]. As early as the year 1998, West and Castenmiller [102] proposed a list of factors that possibly affect the bioavailability and bioconversion of carotenoids and termed it “SLAMENGHI”, representing the species of carotenoids, molecular linkages, amount of carotenoids, food matrices, effects of other nutrients and drugs, nutrient vitamin A status, genetic factors, host-related factors and mathematical interactions. However, in clinical research, the bioavailability of carotenoids is commonly evaluated by the content of carotenoids reaching the systemic circulation after oral administration, without considering their metabolites. In this regard, the present review summarizes the current evidence concerning the dietary strategies for improving carotenoid bioavailability, mainly via “food processing” and “dietary components”. Scheme 2 illustrates the potential mechanisms of bioavailability through assessing the extent of carotenoids released from the food matrix, the efficiency of the incorporation into mixed micelles and the take-up by enterocytes.

In plant tissues, carotenoids are located in the chromoplast, with complex structures inside cells, meaning carotenoids must be first released from the cellular matrix prior to any further absorption. Jeffery et al. [103] demonstrated that the cell walls and chromoplast substructures are the two major barriers for carotenoid release. Given that food processing disrupts the cell walls and membranes and may modify the release of carotenoids, the effects of food processing on carotenoid bioaccessibility have been extensively investigated. Thermal processing and mechanical processing, such as homogenization, high-pressure processing and high-intensity pulsed electric fields, were reported to increase the release of carotenoids to be formed into mixed micelles [104,105,106,107]. Gaston et al. [108] demonstrated that the cell walls of orange fleshed sweet potato were sloughed by traditional thermal processing, which subsequently resulted in an increased bioaccessibility of carotenoids compared to the raw orange fleshed sweet potato. Furthermore, Bengtsson et al. reported that homogenized orange fleshed sweet potato achieved a higher bioaccessibility and they observed a substantial level of cell wall rupture after homogenization [109]. Although food processing opens up the complex structures and subsequently facilitates the liberation of carotenoids, it is worth noting that both thermal and mechanical processing can also lead to adverse impacts [110]. Since carotenoids are sensitive to temperatures and are prone to degradation, thermal treatment often results in substantial loss of carotenoids [111,112]. Moreover, high-pressure homogenization processing (HPP) was reported to decrease the release of lycopene from tomato pulp. The authors speculated that HPP could induce the formation of a strong fiber network and restrain the lycopene to be released [113,114].

In addition to being embedded in chromoplasts, the low bioavailability of carotenoids is also explained by their hydrophobic structures, which make them hard to be incorporated into mixed micelles for subsequent uptake by enterocytes. Given the hydrophobic structures, co-consuming carotenoid-rich foods with dietary lipids is a good dietary strategy to enhance the bioavailability of carotenoids. Numerous in vitro studies have demonstrated an increased extent of carotenoids is incorporated into mixed micelles (described by bioaccessibility or micellarization) with the presence of oils [115,116,117,118]. Additionally, oils rich in unsaturated fatty acids (e.g., canola oil, olive oil, corn oil, soybean oil) were suggested to be superior to oils rich in saturated fatty acids (e.g., palm oil, coconut oil, butter), as evidenced by greater mixed micelle formation [119] and greater uptake by enterocytes [120]. On the other hand, oils rich in long-chain triglycerides were reported to be more effective in solubilizing β-carotene into mixed micelles than oils rich in medium-chain triglycerides [119,121]. Moreover, emerging evidence has indicated that oils prepared in emulsification form result in a better response than the bulk oil [120]. In line with in vitro findings, the favorable effect of co-consuming dietary oils with carotenoid-rich foods was further validated by three RCTs [122,123,124]. Furthermore, in addition to pure oils, several well-designed clinical studies concluded that co-consumption of butter [125], eggs [126] and avocado [127] was also a good dietary strategy for improving carotenoid bioavailability.

## 6. Conclusions

Although the evidence remains controversial, the protective roles of carotenoids in CVDs is suggested, possibly attenuating oxidative stress and mitigating inflammatory response. In addition, the bioavailability of carotenoids should be considered when evaluating the roles of carotenoids in CVD protection. In future research, well-designed RCTs are required, especially concerning their effects on vascular health, including endothelial function and intima–media thickness in populations with impaired vasculature. Moreover, the post-intervention levels of circulating carotenoids should be measured, given that relatively low bioavailability may mask the beneficial effects of carotenoids.
